# Prognostic and predictive implications of Sokal, Euro and EUTOS scores in chronic myeloid leukaemia in the imatinib era—experience from a tertiary oncology centre in Southern India

**DOI:** 10.3332/ecancer.2016.679

**Published:** 2016-10-06

**Authors:** Lakshmaiah Chinnagiriyappa Kuntegowdanahalli, Govind Babu Kanakasetty, Aditi Harsh Thanky, Lokanatha Dasappa, Linu Abraham Jacob, Suresh Babu Mallekavu, Rajeev Krishnappa Lakkavalli, Lokesh N Kadabur, Rudresha Antapura Haleshappa

**Affiliations:** Department of Medical Oncology, Kidwai Memorial Institute of Oncology, Bengaluru, India

**Keywords:** CML, Sokal score, Euro score, EUTOS score

## Abstract

Chronic myeloid leukaemia (CML) is a myeloproliferative disorder. Over the years many prognostic models have been developed to better risk stratify this disease at baseline. Sokal, Euro, and EUTOS scores were developed in varied populations initially receiving various therapies. Here we try to identify their predictive and prognostic implication in a larger population of Indian patients with CML-CP (chronic phase) in the imatinib era.

## Introduction

Chronic myeloid leukaemia (CML) is a myeloproliferative disorder with three different phases at presentation. Early chronic phase disease (CML-CP) is the disease with most favourable outcomes while the advanced accelerated phase (CML-AP) and the blast phase (CML-BP) have poorer outcomes with conventional therapy [1].

Over the years many new developments in management have lead to significant improvements in prognosis of the disease starting from oral or intravenous chemotherapy like busulfan, immunotherapy with interferon alpha (IFNa), and most recently introduction of targeted therapy with oral tyrosine kinase inhibitors (TKI).

Many investigators have tried to develop predictive and prognostic models to risk stratify CML-CP at baseline over the years involving varied treatment strategies and patient populations with the help of varied statistical tests and endpoints. Amongst the many scores available, which one will best predict response to current imatinib therapy and prognosticate survival outcome still remains debatable with very limited data available from the Asian population [2–4]. We here try to assess the most widely used prognostic risk models including Sokal relative risk score, Euro relative risk score, and EUTOS (European Treatment and Outcome Study) score in a large cohort of Indian patients and compare their efficacy as a predictive and prognostic tool in the imatinib era.

## Methods

We identified cases of Philadelphia chromosome (Ph) positive CML-CP from March 2002–February 2015. All the patients had baseline complete blood count, bone marrow examination with conventional cytogenetics and biochemistry. Baseline patient characteristics including age, gender, spleen size, total leucocyte count, platelet count, percentage of myeloblasts, basophils, and eosinophils in peripheral blood were recorded. Sokal score, Euro score, and EUTOS score were calculated according to formulas given below at baseline.

Sokal score = Exp [0.0116 × (age in years - 43.4) + 0.0345 × (spleen size - 7.51) + 0.188 9 ([platelet count ⁄ 700]^2^ - 0.563) + 0.0887 × (blast cell counts - 2.10)], where Exp is the exponential function [5].

Euro score = 0.666 (when age >50 years) + (0.042 × spleen size) + 1.0956 (when platelet count >1500 × 10^9^ ⁄ L) + (0.0584 × blast cell counts) + 0.20399 (when basophil counts >3%) + (0.0413 × eosinophil counts) × 100 [6].

EUTOS score = (7 × basophils) + (4 × spleen size) [7].

All the patients received imatinib 400mg daily as the first line therapy. Patients were monitored for their response or toxicity to imatinib and dose adjustments for toxicities were done as recommended [8]. In case of failure to achieve stated milestones as per ELN 2009 guidelines, patients were eligible to increase daily doses to 800 mg [9]. Responses were defined as previously described by Kantarjian and colleagues [10]. Complete haematologic response (CHR) was defined as a white blood cell count of less than 10 × 10^9^/L, a platelet count of less than 450 × 10^9^/L, no immature cells (blasts, promyelocytes, or myelocytes) in the peripheral blood, and disappearance of all signs and symptoms related to leukaemia, including palpable splenomegaly. Conventional cytogenetics was performed in all cases on bone marrow cells at baseline, and subsequently six monthly to see for cytogenetic response which was identified as: complete cytogenetic response (CCyR), 0% Ph-positive metaphases; partial cytogenetic response (PCyR), 1–35% Ph-positive metaphases; major cytogenetic response (MCyR), 0–35% Ph-positive metaphases; minor, 36–65% Ph-positive metaphases; minimal, 66–95% Ph-positive metaphases; or no response, greater than 95% Ph-positive metaphases, according to ISCN 2013 nomenclature [11–12]. Real-time quantitative PCR (RQ-PCR) was performed on peripheral blood at baseline and after 6–12 monthly to look for molecular response which was defined as major molecular response (MMR) if the BCR-ABL/ABL ratio was < 0.10% on the International Scale [13].

Progression to accelerated phase (CML-AP) was defined as blasts 10–19% in peripheral blood (PB) or bone marrow (BM), basophils at least 20% in PB, persistent thrombocytopaenia unrelated to imatinib therapy, or persistent thrombocytosis unresponsive to imatinib therapy, and evidence of cytogenetic clonal evolution. For persistent grade 3/4 thrombocytopaenia after minimum four weeks of imatinib therapy, lasting beyond 2–4 weeks after drug withdrawal, repeat bone marrow evaluation was done to confirm disease progression. Progression to blast phase (CML-BP) was defined as at least 20% blasts in PB or BM, large foci or clusters of blasts in BM, and/or any extramedullary blast involvement, excluding spleen and liver.

Statistical Package for Social Sciences 20 (SPSS inc., 233 South Wacker Drive, 11th floor, Chicago) was used for analysing the data. Cumulative incidence of CCyR and of MMR, progression-free survival (PFS), and overall survival (OS) were calculated with Kaplan-Meier method [14]. Different risk curves were compared with log-rank test [15].

## Results

We identified 618 cases with CML-CP during the study period. Baseline characteristics for patients are shown in Table 1. Median age at presentation was 35 years with male: female ratio being 1.5:1.

Patient distribution according to risk groups is shown in Table 2. A total of 79 (12.8%) of cases were low risk according to all the three scores while the corresponding number of high risk cases were 47 (7.6%).

Cumulative incidence of CCyR and MMR according to all the three risk scores is shown in Table 3. Cumulative incidence of MMR was 82.3%, 81.7%, and 79.7 % respectively for Sokal, Euro, and EUTOS low risk scores. Difference between various risk groups according to all the three risk scores was statistically significant.

**Figure 1.**
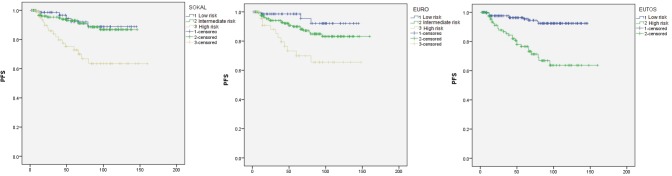
PFS analysis by Kaplan-Meier method.

Kaplan-Meier analysis for PFS is shown in Figure 1. At a median follow-up at 56 months, estimated five years PFS for low, intermediate, and high risk Sokal scores were 92%, 93%, and 73% respectively. Sokal score could not differentiate between those with low or intermediate scores (P = 0.68), however could significantly differentiate the high risk group (P = 0.002 for low versus high risk, P < 0.0001 for intermediate versus high risk). PFS for Euro score was 98%, 90%, and 70% for low, intermediate, and high risk groups respectively. This score also could not differentiate low and intermediate risk groups significantly (P = 0.098), however the differences between low versus high risk (P = 0.001) and intermediate versus high risk (P = 0.006) were statistically significant. Low and high EUTOS scores showed PFS of 97% and 77% respectively with a significant P value of < 0.0001.

**Figure 2.**
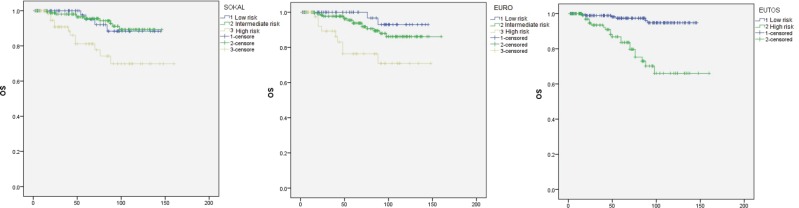
OS analysis by Kaplan-Meier method.

OS analysis for the three risk scores is shown in Figure 2. Estimated five years OS was 95%, 95%, and 81% for low, intermediate, and high risk Sokal groups respectively. Here again the score could not differentiate between low and intermediate risk groups (P = 0.89), but could significantly differentiate the high risk group (P = 0.015 for both low versus high risk and for intermediate versus high risk). The corresponding OS for Euro risk scores was 99%, 94%, and 76%. Difference between low and intermediate risk groups was not statistically significant (P = 0.187). However, differences of that between low versus high (P = 0.002) and intermediate versus high risk (P = 0.005) groups were significant by this score. OS for low and high risk EUTOS scores was also significantly different being 97% and 83% respectively (P < 0.0001).

## Discussion

Attempts to better prognosticate CML-CP, the least aggressive form of the disease, at baseline have provided many variables over the years. Studies as early as in 1970s revealed that Ph negative disease had poorer outcomes than Ph positive disease [16]. Other unfavourable markers proposed included clinical features like fever, marked lymphadenopathy, and skin involvement. Whereaslaboratory parameters included increasing basophilia, myelofibrosis, multiple Philadelphia chromosomes, aneuploidy, muramidasuria, and rising leucocyte alkaline phosphatase values (associated with clinical deterioration) [17].

Attempts to identify the most accurate prediction model has provided us some of the useful risk prediction tools over the years which are summarised in Table 4. Initial systems of scoring, though simpler, had the limitation of small sample size which led to their limited applicability [18–21]. Later, the Sokal score developed in 1984 and the Hasford score developed in 1985 used larger populations for study and became widely applicable. The sophisticated calculators required for these scores are easily available now. However, the major limitation of these scores remains them being primarily validated for patients on busulphan and hydroxyurea (Sokal), or IFNa (Hasford) therapy. This made it imperative to re-evaluate their significance in the current era of TKI based first line therapy, but the results in various studies regarding this validation have been conflicting. EUTOS score is the most recent addition in this group which was specifically developed for patients on TKI treatment. The score is easy to calculate. However its major limitation remains the primary end point of CCyR at 18 months being taken as a predictor of PFS. This is because second generation TKIs can still improve survival outcomes in these cases not achieving CCyR at 18 months, and the most recent ELN guidelines recommend CCyR at six months as an optimal response [23]. EUTOS score has shown to effectively predict risk group at baseline in various studies [24–29]. However some studies have failed to show significant efficacy.

As is evident in Table 2, a variety of western populations were analysed in studies conducted for the Sokal, Euro, and EUTOS scores. Data on Asian population remains limited especially that in Indians. The Asian population supposedly harbours specific polymorphisms that affect sensitivity to TKI [30]. The disease also presents itself at a younger age than in western countries [31]. In our series, the median age at presentation was 35 years. Thus it remains important to assess how well these scores predict outcomes in our predominantly young adult population.

The distribution of cases according to risk groups in our study suggested a predominance of low and intermediate risk groups. This is in accordance with other studies, however proportionate cases with high risk were comparatively more. In the study by Hasford and colleagues, 39%, 37%, and 24% of cases had low, intermediate, and high risk Sokal scores with corresponding Euro scores being 38%, 51%, and 11% [7]. EUTOS score was low for 90% and high for 10% in this study. The Japanese study also had lower proportionate cases of high risk with 18.6%, 8.9%, and 11% cases with Sokal, Euro, and EUTOS high risk [2].

While attempting to address the question of which of the scores predicts best the response to imatinib in our population, we found that all the three scores effectively predicted cumulative incidence of CCyR and MMR. They also significantly prognosticated PFS and OS in our population. While Sokal score and Euro score could not significantly differentiate between low and intermediate risk groups when predicting PFS and OS, they could still significantly differentiate high risk group from low/intermediate risk group. A study in the Nigerian population done by Oyekunle and colleagues also suggested that predictive efficacy for PFS remained poor for Sokal score between the low and intermediate risk groups, however, it could predict difference better between low + intermediate versus high risk groups [32]. Another study from China on the other hand had the limitation of inability to differentiate low and intermediate risk groups reflected in prediction of OS, not PFS [33].

EUTOS score remained most accurate for prognosticating PFS and OS for its two risk groups in our analysis. Various other studies comparing EUTOS score with the previous scores are summarised in Table 5. The two studies from UK and Japan showed inadequate efficacy of EUTOS as a prognostic marker [34, 2]. These studies, however, had a relatively small number of cases classified as EUTOS high risk group, being 31(10%) and 16 (11%) respectively in the two studies. The largest study by Kantarjian and colleagues shows superiority of EUTOS score in imatinib treated European population with similar results in Chinese study [7, 33].

Another important question which we have not addressed here remains if these scores can be used in scenarios where second generation TKIs are planned as first line therapy. Kantarjian and colleagues suggested that nilotinib could improve CCyR and MMR rates at 24 months across all Sokal risk groups in ENESTnd study [35]. Similarly dasatinib improved MMR rates at 24 months across all Hasford risk groups in DASASION study [36]. Jabbour and colleagues suggested that for patients treated with second generation TKIs, EUTOS score could predict CCyR but not MMR [37]. They also found no prognostic effect on survival. Recently bosutinib also showed improved MMR rates at 12 months across all Sokal risk groups in BELA study [38].

Many additional factors might have an impact on outcomes in patients with CML including patient compliance to therapy, racial differences, presence of additional cytogenetic abnormalities (ACA) such as major route abnormalities, altered imatinib pharmacokinetics e.g. OCT-1 transporter activity and imatinib plasma levels, bcr-abl1 mRNA transcript e.g. e13a2 or e14a2 and early treatment responses. It cannot be overemphasised that consideration of all these factors is important while attempting prognostication and prediction of response to treatment in CML patients on TKI therapy. Also using a homogenous end point for response to therapy that also includes the effect of second line TKI as well as using uniform therapy will be essential for future prospective studies in this regard. Further research in this direction remains imperative including use of novel biomarkers that could help in treatment decisions including choice of first line therapy for further improvement in disease outcomes.

Our data confirms the utility of all the three scores in predicting response to imatinib. However, being a dichotomous variable and showing the ability to significantly differentiate between both risk groups in terms of PFS and OS, the EUTOS score appears to outperform as a prognostic model compared to the Sokal and Euro scores in Indian patients in this imatinib era.

## Conclusion

Sokal, Euro, and EUTOS scores have significant predictive efficacy in the Indian population with CML-CP in the imatinib era. However the EUTOS score outperforms as a prognostic model in this scenario. We hope that ongoing research will help us identify better prognostic models to risk stratify patients and tailor therapy according to risk category in the future.

## Conflicts of interest

The authors declare no conflicts of interest.

## Figures and Tables

**Table 1.** Baseline characteristics.

Data Variables	Value
Age, years; median (range)	35 (18–77)
Gender, male : female	1.5:1
Hb, gm/L; median (range)	10.5 (7–18)
Platelet count,10^9^/L; median (range)	399 (130–1300)
Peripheral blasts,%; median (range)	3 (1–9)
Eosinophils, %; median (range)	3 (0–10)
Basophils, %; median (range)	5 (0–13)
LDH, IU/L; median (range)	964 (339–2525)
Spleen, cm; median (range)	10 (0–20)

**Table 2.** Patient distribution according to risk scores in present study.

Risk Group	Sokal Score	Euro Score	EUTOS Score
Low, n (%)	128 (20.7)	125 (20.2)	393 (64.1)
Intermediate, n (%)	355 (57.4)	385 (62.3)	-
High, n (%)	135 (21.8)	108 (17.5)	222 (35.9)

**Table 3.** Predictive efficacy of the three score.

Risk Score	Cumulative Incidence of CCyR (%)	P value	Cumulative Incidence of MMR (%)	P value
(1) Sokal				
Low	88.5	< 0.001	82.3	< 0.001
Intermediate	77		70.4	
High	51		50.8	
(2) Euro				
Low	85	< 0.001	81.7	< 0.001
Inermediate	75.6		69.2	
High	54.5		49.4	
(3) EUTOS				
Low	85.6	< 0.001	79.7	< 0.001
High	55		50.7	

**Table 4.** Risk tools in CML.

Risk Tool	Risk Factors	Risk Groups	a) Population, b) Treatment used	Predictive/Prognostic Implication
Tura *et al* (1981) [18]	Factors: 1. Splenomegaly > 15 cm below costal margin, 2. Hepatomegaly > 6cm below costal margin 3. Thrombocytopaenia < 50x10^9^/l or thrombocytosis > 500x10^9^/l 4. Leucocytosis >100x10^9^/l 5. Blasts in peripheral blood > 1% 6. Promyelocytes and myelocytes peripheral blood > 20%	Stage I (low risk): 0–1 factor Stage II (intermediate risk): 2–3 factors Stage III (high risk): 4–6 factors	a) 255 cases b) Chemotherapy	OS significantly different between three groups (p <0.0005)
Cervantes *et al* (1982) [19]	1. Splenomegaly 2. Hepatomegaly 3. Erythroid precursors in peripheral blood 4. Myeloblasts in bone marrow > 5%	Stage I (low risk): 0–1 factor Stage II (intermediate risk): 2 factors Stage III (high risk): 3–4 factors	a) 121 cases, Spain b) Busulfan	5 year OS Stage I-70%, Stage II-30% Stage III-15%
Kantarjian *et al* (1990) [20]	1. Age ≥ 60 2. Blasts in peripheral blood ≥3% 3. Blasts in bone marrow ≥5% 4. Basophils in peripheral blood ≥7% 5. Basophils in bone marrow ≥3% 6. Platelet count ≥700 x10^9^/L 7. Splenomegaly ≥10 cm below costal margin Accelerated phase: a) Blasts in peripheral blood ≥ 15% b) Basophils in peripheral blood ≥ 20% c) Blasts and promyelocytes in peripheral blood ≥30% d) Platelet count ≤100x10^9^/L e) Cytogenetic clonal development	Stage I: 0–1 factor Stage II: 2 factors Stage III: 3 or more factors Stage IV: accelerated phase	a) 406 cases b) Chemotherapy	Median OS Stage I-56 months Stage II-45 months Stage III-30 months Stage IV-30 months
Kantarjian *et al* (1985) [21]	1. Circulating basophils 2. Basophils in bone marrow 3. Race 4. Age 5. Additional chromosome abnormalities	Low risk- HR < 0.8 Intermediate risk- HR 0.8 to 1.39 High risk- HR > 1.39	a) 303 cases b) Busulphan or hydroxyurea or OAP (vincristine, cytarabine, prednisolone) + anthracycline or cyclophosphamide or splenomegaly	Median OS: Low risk- 53 months Intermediate risk- 39 months High risk- 25 months
Sokal score (1984) [5]	1. Age 2. Spleen size below costal margin (cm) 3. Platelet count 4. Blasts in peripheral blood (%)	Low risk: < 0.8 Intermediate risk: 0.8–1.2 High risk: > 1.2	a) 813 cases, Europe, USA b) Busulfan or hydroxyurea	OS at two years: Low risk- 90% High risk- 65%
Hasford score (1996) [22]	1. Age 2. Spleen size below costal margin (cm) 3. Erythroblasts in peripheral blood (%) 4. Eosinophils in peripheral blood (%) 5. Gender	Low risk: < 1.4 Intermediate risk: 1.4–2.0 High risk: > 2.0	a) 490 cases, Germany b) Busulphan, hydroxyurea, IFNa	Five years OS: Low risk- 90%
Euro score (1998) [6]	1. Age 2. Spleen size below costal margin (cm) 3. Blasts in peripheral blood (%) 4. Eosinophils in peripheral blood (%) 5. Basophils in peripheral blood (%) 6. Platelet count	Low risk: ≤ 780 Intermediate risk: > 780 ≤ 1480 High risk: > 1480	a) 1303 cases, Europe, Japan, USA b) IFNa	Median OS: Low risk- 98 months Intermediate risk- 65 months High risk-42 months
EUTOS score (2011) [7]	1. Basophils in peripheral blood (%) 2. Spleen size below costal margin (cm)	Low risk: ≤ 87 High risk: > 87	a) 2060 cases,Europe b) TKI (imatinib 400mg/d in 41% cases, imatinib 400mg/d +LDAC or IFNa in 34% cases, imatinib 600–800mg/d in 25% cases	CCyR at 18 months: Low risk-86% High risk-66% PFS at five years: Low risk- 90% High risk- 82%

IFNa- interferon alpha, LDAC- low dose cytosine arabinoside

**Table 5.** Comparison studies of Sokal, Euro and EUTOS scores in imatinib era.

Reference	Predictive Implication	Prognostic Implication
Marin *et al* (UK) [34]	EUTOS not predictive of CCyR, MMR. Sokal has predictive efficacy.	EUTOS not prognostic of PFS, OS. Sokal has prognostic efficacy.
Yamamoto *et al* (Japan) [2]	EUTOS not predictive of CCyR or MMR. Sokal and Euro have predictive efficacy.	EUTOS not prognostic of EFS, PFS, OS. Sokal and Euro have prognostic efficacy.
Hasford *et al* (Europe) [7]	EUTOS better predictive of CCyR. Sokal or Euro do not have predictive efficacy.	EUTOS better prognostic of PFS. Sokal or Euro do not have prognostic efficacy.
Tao *et al* (China) [33]	EUTOS better predictor of CCyR. Sokal and Euro unable to differentiate intermediate Vs high risk for CCyR.	EUTOS better prognostic of PFS and OS. Sokal unable to differentiate low Vs intermediate risk for OS. Euro unable to differentiate intermediate Vs high risk for PFS and OS.
Present study (India)	EUTOS, Sokal, and Euro scores predictive of cumulative incidence of CCyR and MMR	EUTOS better predictor of PFS and OS. Sokal and Euro unable to differentiate low and intermediate risk for PFS and OS.

## References

[1] Galton DAG (1969). Chemotherapy of chronic myelocytic leukemia. Sem Hematol.

[2] Yamamoto E (2014). European treatment and outcome study score does not predict imatinib treatment response and outcome in chronic myeloid leukemia patients. Cancer Sci.

[3] Yahng SA (2014). Prognostic discrimination for early chronic phase chronic myeloid leukemia in imatinib era: comparison of Sokal, Euro, and EUTOS scores in Korean population. Int J Hematol.

[4] Than H (2012). The EUTOS score is highly predictive for clinical outcome and survival in Asian patients with early chronic phase chronic myeloid leukemia treated with imatinib. Blood.

[5] Sokal JE (1984). Prognostic discrimination in “good-risk” chronic granulocytic leukemia. Blood.

[6] Hasford J (1998). A new prognostic score for survival of patients with chronic myeloid leukemia treated with interferon alfa. Writing Committee for the Collaborative CML Prognostic Factors Project Group. J Natl Cancer Inst.

[7] Hasford J (2011). Predicting complete cytogenetic response and subsequent progression-free survival in 2060 patients with CML on imatinib treatment: the EUTOS score. Blood.

[8] Deininger MW (2003). Practical management of patients with chronic myeloid leukemia receiving imatinib. J Clin Oncol.

[9] Baccarani M (2009). Chronic myeloid leukemia: an update of concepts and management recommendations of European LeukemiaNet. J Clin Oncol.

[10] Kantarjian H (2002). Hematologic and cytogenetic responses to imatinib mesylate in chronic myelogenous leukemia. N Engl J Med..

[11] Baccarani M, Pane F, Saglio G (2008). Monitoring treatment of chronic myeloid leukemia. Haematologica.

[12] Shaff er LG (2013). An International System for Human Cytogenetic Nomenclature.

[13] Hughes T (2006). Monitoring CML patients responding to treatment with tyrosine kinase inhibitors: review and recommendations for harmonizing current methodology for detecting BCR-ABL transcripts and kinase domain mutations and for expressing results. Blood.

[14] Kaplan GL, Meier P (1958). Nonparametric estimation from incomplete observations. J Am Stat Assoc.

[15] Peto R, Pike MC (1973). Conservation of the approximation ı(O - E)2/E in the log rank test for survival data on tumor incidence data. Biometrics.

[16] Ezdinli EA (1970). Philadelphia-chromosome-positive and negative chronic myelocytic leukemia. Ann Intern Med.

[17] Theologides A (1972). Unfavourable signs in patients with chronic myelocytic leukemia. Ann Intern Med.

[18] Tura S, Baccarani M, Corbelli G (1981). Staging of chronic myeloid leukaemia. Br J Haematol.

[19] Cervantes F, Rozman C (1982). A multivariate analysis of prognostic factors in chronic myeloid leukemia. Blood.

[20] Kantarjian HM (1990). Proposal for a simple synthesis prognostic staging system in chronic myelogenous leukemia. Am J Med.

[21] Kantarjian HM (1985). Chronic myelogenous leukemia: a multivariate analysis of the associations of patient characteristics and therapy with survival. Blood.

[22] Hasford J (1996). Analysis and validation of prognostic factors for CML. German CML Study Group. Bone Marrow Transplant.

[23] Baccarani M (2013). European LeukemiaNet recommendations for the management of chronic myeloid leukemia. Blood.

[24] Uz B (2013). EUTOS CML prognostic scoring system predicts ELN-based ‘event-free survival’ better thanEuro/Hasford and Sokal systems in CML patients receiving front-line imatinibmesylate. Hematology.

[25] Tiribelli M (2013). EUTOS score predicts long-term outcome but not optimal response to imatinib in patientswith chronic myeloid leukaemia. Leuk Res.

[26] Hoffmann VS (2013). The EUTOS prognostic score: review and validation in 1288 patients withCML treated frontline with imatinib. Leukemia.

[27] Breccia M (2012). The EUTOS score identifies chronic myeloid leukeamia patients with poor prognosis treated with imatinib first or second line. Leuk Res.

[28] Tiribelli M (2012). EUTOS scoreidentifies cases with poor outcome in patients with early chronic phase chronic myeloid leukemia, though not predictive for optimal response to imatinib. In:ASH Annual Meeting Abstract.

[29] Bonifacio M (2014). EUTOS score predicts early optimal response to imatinib according to the revised 2013 ELN recommendations. Ann Hematol.

[30] Singh O (2012). SLC22A1-ABCB1 haplotype profiles predict imatinib pharmacokinetics in Asian patients with chronic myeloid leukemia. PLoS ONE.

[31] Bansal S, Prabhash K, Parikh P (2013). Chronic myeloid leukemia data from India. Indian J Med Paediatr Oncol.

[32] Oyekunle AA (2012). The predictive value of the Sokal and Hasford scoring systems in chronic myeloid leukaemia in the imatinib era. J Hemat Malignancies.

[33] Tao Z (2014). EUTOS score predicts survival and cytogenetic response in patients with chronic phase chronic myeloid leukemia treated with first-line imatinib. Leuk Res.

[34] Marin D, Ibrahim AR, Goldman JM (2011). European Treatment and Outcome Study (EUTOS) score for chronic myeloid leukemia still requires more confirmation. J Clin Oncol.

[35] Kantarjian HM (2011). Nilotinib versus imatinib for the treatment of patients with newly diagnosed chronic phase, Philadelphia chromosome-positive, chronic myeloid leukaemia: 24-month minimum follow-up of the phase 3 randomised ENESTnd trial. Lancet Oncol.

[36] Kantarjian HM (2012). Dasatinib or imatinib in newly diagnosed chronic-phase chronic myeloid leukemia: 2-year follow-up from a randomized phase 3 trial (DASISION). Blood.

[37] Jabbour E (2012). EUTOS score is not predictive for survival and outcome in patients with early chronic phase chronic myeloid leukemia treated with tyrosine kinase inhibitors: a single institution experience. Blood.

[38] Cortes JE (2012). Bosutinib versus imatinib in newly diagnosed chronic-phase chronic myeloid leukemia: results from the BELA Trial. J Clin Oncol.

